# Validation of Genetic Markers Associated to Oxygen Availability in Low-Grade Copper Bioleaching Systems: An Industrial Application

**DOI:** 10.3389/fmicb.2019.01841

**Published:** 2019-08-09

**Authors:** Mayra Cortés, Sabrina Marín, Pedro Galleguillos, Dina Cautivo, Cecilia Demergasso

**Affiliations:** ^1^Centro de Biotecnología Profesor Alberto Ruiz, Universidad Católica del Norte, Antofagasta, Chile; ^2^Centro de Investigación Científico Tecnológico para la Minería, Antofagasta, Chile

**Keywords:** *Leptospirillum ferriphilum*, heap bioleaching, oxygen availability, electron transport chain, genetic markers, industrial monitoring

## Abstract

Forced aeration is one of the major energy consumption factors of the bioleaching process of run-of-mine ore. The effect of aeration in the microbial community has scarcely been studied at industrial level. *Leptospirillum ferriphilum* is one of the most representative species of the Fe^3+^ producing population in this kind of systems. We analyzed the effect of oxygen availability on *L. ferriphilum* by growth activity and transcriptional dynamics of its two terminal oxidases (cbb3 and bd complexes) under different experimental test: culture reactor, bioleaching column, and industrial heap tests. Relatively low O_2_ availability triggered important changes in the microbial community composition, cell growth, microbial activity and *cydAB* genes transcription in all cases of study. We assessed the potential role of the terminal oxidases on the adaptation to variable aeration conditions in different lifestyles of *L. ferriphilum* and identified transcriptional markers associated to oxygen metabolism in an industrial system. An interesting hypothesis about the possible role of the cbb3 complex in the response to oxidative stress as well as their role as a high oxygen-affinity oxidase in *L. ferriphilum* is proposed and discussed. This study successfully proves the function of the *cydAB* genes as valid genetic markers for low-grade copper industrial bioleaching systems.

## Introduction

It is known that forced aeration is the main O_2_ and CO_2_ source in bioleaching systems, both sustaining microbial activity and subsequent metal recovery (Ghorbani et al., 2016; Lakshmanan et al., 2016). Nowadays it is acknowledged that forced aeration is estimated to account for 30% of the cost of energy in a copper bioleaching process. However, it is not possible to measure the gas availability through the entire ore-heap as it implies a huge economic cost. Actually, the operation practices regarding aeration includes only switching on or off the blowers systems in the process and it is not possible to know the real effect of these changes on the microbial activity and metal recovery. For this reason, a current biotechnological and operational challenge is to identify an effective and reliable method that allows monitoring O_2_ and CO_2_ availability in industrial bioleaching process as well as their effect on the microbial consortia.

Dissolved oxygen (DO) is the main final electron acceptor in most bioleaching microorganisms (aerobic; chemolithoautotrophic) for energy generation by iron (II) and/or reduced sulfur oxidization pathway (Levicán et al., 2012). Prokaryotic cells have the ability to induce branched-respiratory chains with multiple oxidases that, depending on species, can have different affinities for oxygen (Bueno et al., 2012). This ability provides them with a flexible respiratory system able to adapt to variable oxygen availability by modulating gene expression as a function of the oxygen levels (Levicán et al., 2012). This trait confers an extraordinary adaptive ability to microorganisms to both colonize different environments and to opportunely respond to oxygen variations (Bueno et al., 2012).

Several works have described the diversity of species inhabiting bioleaching systems and acid mine drainage environments and their relevance in the metal recovery process (Bond and Banfield, 2001; Demergasso et al., 2005; Xie et al., 2007; Remonsellez et al., 2009; Bobadilla-Fazzini et al., 2017). *Leptospirillum ferriphilum* is a Gram-negative and extreme acidophilic bacterium that can derive energy only from ferrous iron oxidization and can only use oxygen as final electron acceptor (Coram and Rawlings, 2002). This species is recognized as one of the most stable and abundant microbial bacterium of bioleaching systems (Demergasso et al., 2005; Galleguillos et al., 2009; Remonsellez et al., 2009) because of its wide temperature tolerance range (37–48°C) (Acosta et al., 2014), its high Fe^2+^ affinity and its high Fe^3+^ tolerance (Coram and Rawlings, 2002). These traits make it also one of the key player in the industrial bioleaching processes (Galleguillos et al., 2013).

It has been proposed that species of the *Leptospirillum* genus probably have the ability to flexibilize their cellular respiration in response to changes in oxygen-tension (Levicán et al., 2012), but the mechanisms underlying this ability are still unknown. Direct effect of DO availability in the performance of biomining microorganisms has scarcely been studied and the available knowledge is more related to the effect of oxygen on metal recovery (Cautivo and Gentina, 2009) than to microbial growth and activity.

It is known that under O_2_-limited conditions, some other bacteria induce high-oxygen-affinity cytochrome oxidases to respire the traces of gas (Bueno et al., 2012). The cbb3-type (Hemme-Copper Oxidase family, Type A) and bd-type (Quinol Oxidase Family only found in bacteria and archaea) oxidases have been described as cytochromes with high-oxygen-affinity in several species, such as *Escherichia coli, Rhodobacter capsulatus*, and *Bradyrhizobium japonicum* (Borisov et al., 2011; Bueno et al., 2012). Additionally, in *E. coli*, the bd-type cytochrome has been associated to protection against oxidative stress (Lindqvist et al., 2000). In bioleaching species, there is one study related to this topic and was performed on the most studied acidophilic species, *Acidithiobacillus ferrooxidans*, that has terminal cytochrome *c* oxidase aa3-type and terminal cytochrome bd and bo-type ubiquinol oxidases (Brasseur et al., 2004). The authors observed that *A. ferrooxidans* synthesizes cytochrome bd oxidase in highly aerated cultures and suggested that this oxidase probably plays an active role in the rapid consumption of oxygen for maintaining a low intracellular oxygen concentrations compatible with nitrogenase function. Analysis performed on *Leptospirillum* genomes showed that all species from this genus presented both cbb3 and bd-type terminal oxidases (Levicán et al., 2012) and, unlike *A. ferrooxidans, Leptospirillum* did not have cytochromes with recognized low-oxygen-affinity. Levicán et al. (2012) inferred, through a genomic analysis, that *Leptospirillum* sp. would be able to sustain a high iron oxidization rate even at low oxygen levels due to the presence of the high-oxygen-affinity cbb3 cytochrome, which represents an adaptive advantage for the genus in the system.

It has been reported that expression of genes associated to cytochromes with high-oxygen-affinity like cbb3 and bd-type oxidases is strongly induced in cells grown under microaerobic or anaerobic conditions (Pitcher and Watmough, 2004), while those associated to cytochromes with low-oxygen-affinity, as cytochrome bo3 ubiquinol oxidase, are expressed in cells grown under aerobic conditions in *E. coli* (Pitcher and Watmough, 2004). Parro et al. (2007) mentioned an induction of the operon *cydAB* in *Leptospirillum ferrooxidans* from one site (S1) of the Tinto River having a relatively lower oxygen availability (13.1% sat O_2_ vs. 64.8% sat O_2_), suggesting that bd-type oxidase would have a high-affinity for oxygen in this species. However, the transcriptomic data of *cydAB* was not properly described in that study so results are inconclusive.

Omics information of chemolithoautotrophic and acidophilic species has been increasing through time (Orellana and Jerez, 2011; Bonnefoy and Holmes, 2012; Aliaga et al., 2013, 2015; Quatrini and Johnson, 2018). However, comprehension of the metabolic response of these microorganisms to the environmental variability inherent to bioleaching processes is still scarce. The aim of this research was to study the transcriptomic response of the terminal cytochrome cbb3 and bd oxidases in *L. ferriphilum* growing under different laboratory and industrial environments and different oxygen availabilities with the biotechnological purpose to identify and validate molecular markers to monitor the aeration parameter in industrial bioleaching processes.

## Materials and Methods

### Cultures of *Leptospirillum ferriphilum*

*Leptospirillum ferriphilum* type-strain DSM 14647 was grown in 3 L reactors with 1 L of ABS medium (g/L), (NH_4_)_2_SO_4_ (0.15), Na_2_SO_4_ (0.066), KCl (0.05), MgSO_4_^∗^7H_2_O (0.5), KH_2_PO_4_ (0.05), Ca(NO_3_)_2_^∗^4H_2_O (0.014), trace element (Bryan et al., 2012), and with FeSO_4_ 7H_2_O (50 mM) at 37°C, pH 1.5, agitation 200 rpm and three different conditions: 3, 10, and 23% (control) of O_2_ in the inlet air, respectively. Oxygen concentration was regulated by gas mixture (atmospheric compressed air and N_2_) and feeding flow rate at 400 mL/min in the inlet flow. Experiment was reproduced to obtain an experimental repetition. The input and output air O_2_ concentration (%) in the reactors and the DO concentration of the cultures were monitored using a Micro-Oxymax respirometer (Columbus Instruments) and a portable DO meter, respectively. Cell growth curves were built with data of cell count in Neubauer camera every 3 h for 3 days. All cultures were sampled at the late exponential growth phase when ferrous consumption reached ∼90% and cells were in abundance and metabolically active. Cell concentrations of reactors at the sampling time were in the range of 9.0 × 10^7^ to 1.5 × 10^8^ cell/mL in both experiments. Cells were recovered by vacuum filtration through nitrocellulose membrane (0.2 μm), and were immediately preserved at −80°C in RNAlater^®^ solution (Invitrogen ^TM^ AM7021) until nucleic acid isolation.

### Bioleaching Column Tests

**FIGURE 1:**
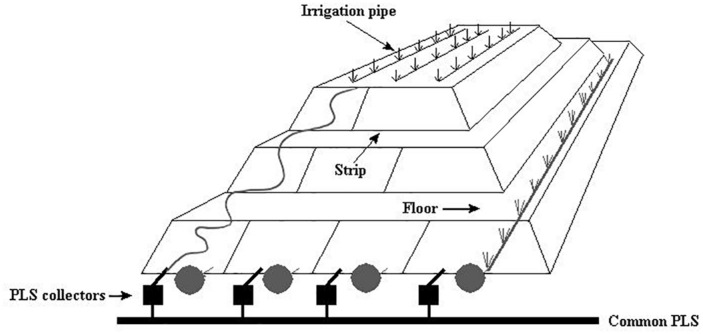
Schematic view of heap bioleaching at the Escondida mine adapted from Acosta et al. (2014).

Two copper bioleaching columns (col1/control and col2/air-test, 1 m high and 0.6 m in diameter) were simultaneously run and controlled. Samples were collected at different operational days (OD) (Table 1) for population and transcriptomic analysis between February and March 2015. Columns were operated at an average temperature of 21–25°C in an open circuit system fed with raffinate and inoculated with a microbial consortium from the Escondida Mine (MEL) industrial bioleaching heap, located 170 km south-east of Antofagasta, Chile (Figure 1). All physicochemical parameters (pH, temperature, nutrient-NH_4_) were maintained stable during the operation except the inlet air of col2 that was intentionally interrupted two times by air displacement with N_2_ (Table 1). The percentage of inlet-oxygen, when it was turned on, was constantly 21%. Input (-in) and output (-out) O_2_ concentration (percentage of O_2_ in the air) were continually monitored in both columns by a Micro-Oxymax respirometer (Columbus Instruments). Cell collection was performed before and after the input-airflow interventions of col2 by filtration of 40 L of PLS (Pregnant Leach Solution) through a 0.2 μm nitrocellulose membranes. Cells were preserved as described in Section “Cultures of *Leptospirillum ferriphilum*.”

**TABLE 1:** Samples from *L. ferriphilum* cultures, bioleaching columns and industrial heap with details of their respective air (O_2_) feeding situation.

**Sample**	**Identification label**	**Aeration condition**
Reactor Exp-1	3% E1	3% of inlet-O_2_ (DO 0.3 mg/L)
Reactor Exp-1	10% E1	10% of inlet-O_2_ (DO 1 mg/L)
Reactor Exp-2	3% E2	3% of inlet-O_2_ (DO 0.3 mg/L)
Reactor Exp-2	10% E2	10% of inlet-O_2_ (DO 1 mg/L)
Reactor Exp-2	23% E2	23% of inlet-O_2_ (DO 6.5 mg/L)
col1	OD50	
col2	OD62^(*)^	10.68% O_2_-out
col2	OD64	0.19% O_2_-out
col2	OD69^(**)^	2.55% O_2_-out
col2	OD76	15.64% O_2_-out
col2	OD90^(*)^	11.81% O_2_-out
col2	OD91	1.88% O_2_-out
col2	OD97	0% O_2_-out
col2	OD104	0% O_2_-out
Strip 317	S317	Permanently aerated
Strip 318	S318	Permanently aerated
Strip 405	S405	Permanently non-aerated
Strip 410	S410 A	Blower turn-on T0
Strip 410	S410 B	Blower turn-off T1
Strip 410	S410 C	Blower turn-off T2
Strip 410	S410 D	Blower turn-off T3

*(^∗^) Air displacement with N_2_ in col2. (^∗∗^) Restoration of normal aeration in col2. OD, operational day of the column; O_2_-out, out-put oxygen concentration; T, time; Exp, experiment; DO, dissolved oxygen in the culture media.*

### Industrial Test and Samples

The industrial bioleaching heap from Escondida Mine was built with ROM (run of mine) material ore characterized as low-grade sulfide minerals (Soto et al., 2013; Acosta et al., 2014). This heap is divided into lifts and strips. The strips are irrigated with raffinate which percolates vertically up to the bottom, finally obtaining the PLS (Figure 1). Six PLS samples from four strips (317, 318, 405, and 410), each with different aeration conditions, were sampled (Table 1). Briefly, PLS (40 L) of strips 317 and 318 (permanently aerated, with both blowers in turn-on) and 405 strip (permanently non-aerated, with blowers in turn-off) were obtained in a first sampling. In addition, PLS (40 L) from the aerated strip 410 was obtained before (one sample) and after (three samples) an aeration interruption. Aeration suspension was carried out by turning off the blowers (forced aeration) located in the base of the MEL heap. All cell samples were collected and preserved as described above.

### Microbial Community Analysis

Genomic DNA from cells was isolated from eight column samples (OD 62, 64, 69, 76, 90, 91, 97, and 104) and seven industrial samples (S317, 318, 405, 410A, 410B, 410C, and 410D) (Table 1) as previously described (Demergasso et al., 2005). Effect of aeration on bioleaching community and population dynamics were determined by absolute quantification of 16S rRNA genes associated to nine acidophilic species using quantitative Real-Time PCR (q-PCR) as previously described (Marín et al., 2017).

### RNA Purification and cDNA Synthesis

RNA of all samples (Supplementary Table 1) was isolated and purified as previously described (Marín et al., 2017). Reverse transcription was performed from digested (RQ1 RNase- Free DNase, Promega M 6101) total RNA using the First Strand cDNA Synthesis Kit (Thermo Scientific, cat. No. K1612) in presence of ribonuclease inhibitor RNasin^®^ (Promega, N2511) and random primers, following directions provided by the manufacturer. Retrotranscripts were stored at −20°C for 1 week or −80°C for longer time.

### Candidate Genes and Primer Design

The metagenomic analysis of a mix of industrial bioleaching samples from MEL was previously performed (Acosta et al., 2017). *L. ferriphilum* related sequences were separated trough mapping with CLC Genomic Workbench 8.1 (QIAGEN Bioinformatics) against *Leptospirillum* genomes. A representative reconstructed genome was chosen based on the coverage and the depth of the sequencing. The sequences of the reconstructed genome were then annotated using the RAST Server (Aziz et al., 2008).

Primers were designed for genes coding proteins related to terminal oxidases cbb3 and bd from *L. ferriphilum* (Table 2). In addition, primers were designed for 16S rRNA and *alaS* genes from *L. ferriphilum*. These housekeeping genes were used as endogenous controls for the gene expression calculation. The chosen genes of *L. ferriphilum* strains ML04 (genome paper), SpCl (genome paper) and the reconstructed MEL genome were aligned with ClustalW (Larkin et al., 2007) and conserved regions were used as base for the primer design. Finally, the promissory candidate primers were evaluated with Primer-BLAST (Ye et al., 2012).

**TABLE 2:** Primer sequences used to relative expression analysis in *L. ferriphilum.*

**Primer**	**Primer sequence (5′–3′)**	**Annealing temperature (°C)**	**Efficiency (E)**
16SF	TAC AAG CTT CCG CTC CTG	60	1.80
16SR	CCG GGC AAA AGT GGT TTA CA	60	1.80
alaSF	TAC CCG GAG CTT AGA ACA TC	60	2.06
alaSR	TAT CGA GCG GAA ATC CAT GC	60	2.06
cbb3F	CCA TCA AGG GGT GGC ATC TG	60	2.15
cbb3R	GGG CAT CCA GGT CCT TTT TC	60	2.15
cydBF	CGA TTC GAT CCT GTC ACC ATG C	60	2.0
cydBR	CGT GAC ATA ACC GGC GCT GA	60	2.0
cydAF	TGA TTC TGG TGC TGT GGT CC	60	2.17
cydAR	CGA CAA AGA GTC CCC AGT CC	60	2.17

All primers were validated in their specificity, repeatability and sensibility (Table 2, Supplementary Figures 1–5, and Supplementary Table 1). Pearsons (R) coefficients of 0.99, optimal efficiencies (E) (100–110%) and dynamic ranges of 1 × 10^2^–1 × 10^7^ were obtained for all selected primers (Table 2).

### Relative Expression Analysis by RT-qPCR

Relative gene expression analysis was performed by RT-qPCR (two steps) using a real-time PCR machine Rotor-Gen Q (Qiagen). Amplification reaction was composed by: 1X SensiMix^TM^ SYBR No-ROX (BIOLINE, cat. No. QT650-05), 0.1 μM of each specific primer designed for selected genes (Table 2), 1 μL of previously generated cDNA as template and water to complete a final volume of 10 μL. The thermal cycling protocol was as follows: initial denaturation at 95°C for 10 min followed by 40 cycles at 95°C for 30 s, 60°C for 30 s, and 70°C for 30 s. Each qPCR run was performed using technical triplicates. Relative quantification of transcripts was calculated by Pfaffl mathematical model (Pfaffl, 2001) using Excel and REST© 2009 software (Pfaffl et al., 2002). Pfaffl model has advantage to consider the real Efficiency (E) value of qPCR reaction in the calculation unlike other available models such as 2^–ΔΔ^ ^CQ^ (Livak and Schmittgen, 2001). Pfaffl model has been successfully used in previous gene expression analysis for acidophilic microorganisms (Galleguillos et al., 2013; Acosta et al., 2014; Marín et al., 2017).

It is known that to use one or more validated reference genes is crucial to obtain reliable and repeatable relative expression data (Vandesompele et al., 2002). In this work, all relative gene expression analyses were normalized using two reference genes previously validated by geNorm (Vandesompele et al., 2002) and NormFinder (Andersen et al., 2004) algorithms: *alaS* and *16S* rRNA. The relative expression data was represented as Log_2_ (ratio). All RT-qPCR analyses performed in this work presented negative controls without amplification. Significant differences in relative expression were adjusted at 1 < Log_2_-ratio < −1. In other words, all data (Log2-ratio) > −1 and < 1 was considered as a non-significant difference of relative gene expression.

## Results

RNA concentrations obtained from samples of the pure cultures, experimental column and MEL strips ranged between 26.4 and 173.0 ng/μL (Supplementary Table 1). Efficiency obtained for each primer set was between 1.80 and 2.17 (Table 2), and high specificity (Supplementary Figures 1–5) and sensitivity (results not shown) for designed primers were achieved in all cases.

### Effect of O_2_ Availability on *Leptospirillum ferriphilum* DSM 14647 and the Microbial Bioleaching Community

**FIGURE 2:**
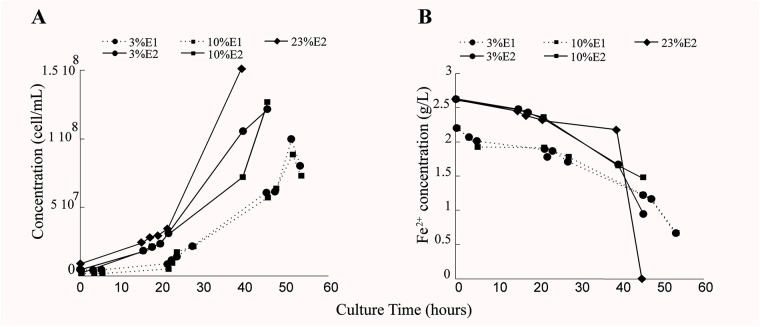
Cell growth (cell/mL) **(A)** and ferrous consumption (g/L) **(B)** of *Leptospirillum ferriphilum* DSM 14647 under the analyzed conditions (3, 10, and 23% of inlet-O_2_). The dotted and continuous lines are showing the results of the experimental repetitions E1 and E2, respectively.

Growth of *L. ferriphilum* DSM 14647 changed depending on the analyzed O_2_ conditions. Less cell growth was observed in reactor with 3% (0.3 mg/L of DO in the culture medium) and 10% (1 mg/L of DO in the culture medium) of inlet-O_2_ with respect to the control condition with 23% of inlet-O_2_ (6.5 mg/L of DO in the culture medium) (Figure 2A). In addition, differences in the time needed to reach the late exponential growth phase and to complete ferrous oxidization between the three O_2_ conditions analyzed was observed (Figures 2A,B). Although the growth and ferrous oxidizing activity of *L. ferriphilum* were not identical in both experimental repetitions, a similar tendency in the repetitions growing at 3 and 10% of inlet-O_2_ was observed (Figures 2A,B). *L. ferriphilum* reached the higher cell growth and complete ferrous oxidization at 40 h of cultivation in the control (23% O_2_), while it was unable to completely oxidize the substrate at O_2_ restricted incubation (3 and 10% of inlet-O_2_) up to 45 and 51 h of cultivation, respectively (Figures 2A,B).

**FIGURE 3:**
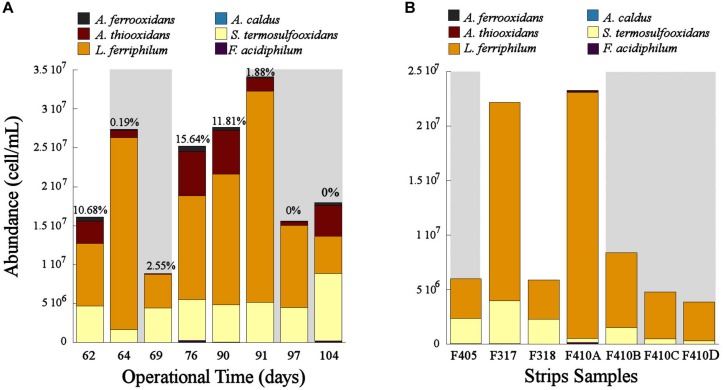
Population dynamics of the microbial community in the bioleaching column **(A)** and in the industrial bioleaching strips **(B)** in response to variable O_2_ availabilities. Values above each bar shows the O_2_-out level in the different operational days of the bioleaching column. Gray area shows the samples with low O_2_ availability **(A)** and the strips with interrupted aeration **(B)**.

The population dynamics of the microbial consortia in the experimental columns and the industrial strips showed that *L. ferriphilum* was the predominant species in these systems (Figure 3). However, the cell concentration changed depending on the aeration. In the bioleaching column, the cell concentration decreased when the aeration was suspended (OD62, 69, 97, and 104) and increased when the aeration was restored (OD76, 90, and 91) (Figure 3A). Moreover, a significant decrease of *L. ferriphilum* concentration after the second aeration suspension in OD90 was noticed. Even lower concentrations of *L. ferriphilum* in the bioleaching column were observed at OD 69 and 104, after 7 and 14 days of aeration suspension respectively (Figure 3A). The same tendency was observed in samples of the industrial bioleaching heap where most of non-aerated strips (S405, S410B, S410C, S410D) presented lower cellular concentration compared to aerated strips (S317, S410A) (Figure 3B and Table 1).

### Identification and Industrial Validation of Transcriptional Markers Associated With Oxygen Availability in a Bioleaching System

**FIGURE 4:**
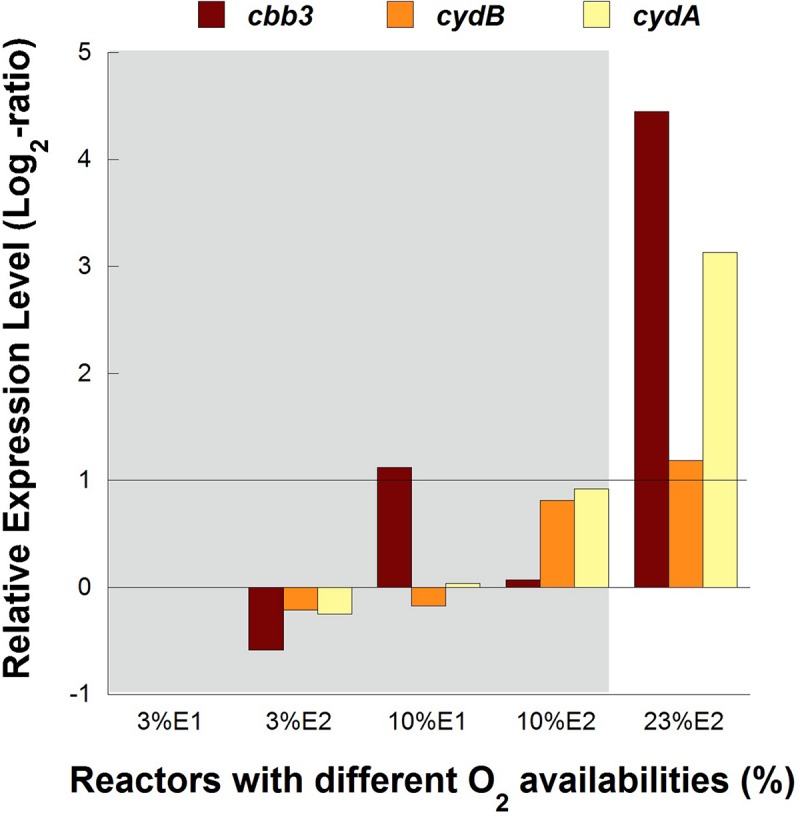
Transcription dynamics of *cbb3*, *cydA*, and *cydB* genes in *L. ferriphilum* DSM 14647 growing in pure cultures with different O_2_ concentrations in the air. Gray area shows the reactors with lower O_2_ levels.

No significant differences (1 > Log_2_ ratio > −1) in transcription levels of *cbb3* and *cydAB* genes were observed between cultures at 3 and 10% of O_2_ in both experimental replicates of pure cultures, while both cytochrome complexes were overexpressed 4.5 and 3.3 folds, respectively, in the control reactor with 23% O_2_ in the feed (Figure 4).

**FIGURE 5:**
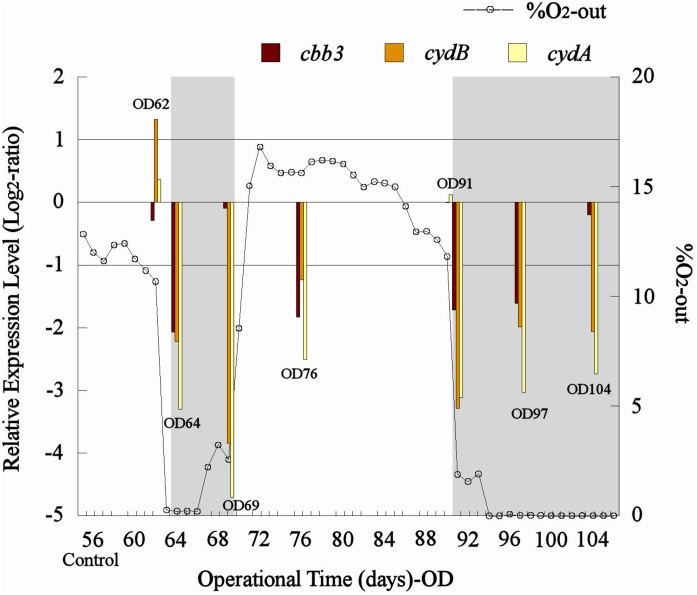
Transcriptional dynamics of *cbb3*, *cydA*, and *cydB* genes of *L. ferriphilum* during aeration interventions in the bioleaching column. OD, operational day of the column. The dotted line shows the O_2_-out (%) from col2. Gray area shows periods when aeration was interrupted in the column. When underexpressed bars are approaching zero, that means that gene transcription is increasing.

A drop in the percentage of O_2_-out (from approximately 11 to 0%) in the bioleaching column test (col2) was recorded after both aeration interruption events (days 62–66 and 90–104, respectively) (Figure 5). Also, similar transcription levels (1 > Log_2_ ratio > −1) were observed for the control sample and the samples taken before the aeration interruption (OD62) in col2 for all analyzed genes, and then an abrupt decrease in their transcription (Log_2_ ratio < −1) was evidenced after O_2_ displacement with N_2_ (OD64 and OD91) (Figure 5). The *cbb3* gene showed a greater transcription level (observed as a minor underexpression with respect to the control) than *cydAB* genes under limited O_2_ conditions (OD64, 69, 91, 97, and 104) (Figure 5), while all three genes tended to restore their transcription levels after the aeration was restarted in the column (Figure 5). Although the *cbb3* gene did not show transcriptional dynamics clearly related to oxygen availability, an evident increase of their transcription (perceived as a gradual decrease in their underexpression through time) after 7 and 13 days of exposition to low O_2_ availability (OD69 and OD104, respectively) was observed (Figure 5). This result differs from that observed in *L. ferriphilum* DSM 14647 pure cultures where the *cbb3* gene was overexpressed in the highest O_2_ condition (23%) (Figure 4). This increment in transcription apparently induced by low oxygen levels in col2 was not observed in *cydAB* genes, which maintained their underexpression (with respect to the control) along all the period time when aeration was suspended in the col2 (OD64, 0.19% O_2_; OD69, 2.55% O_2_; OD91, 1.88% O_2_; OD97, 0% O_2_; and OD104, 0% O_2_) (Figure 5).

**FIGURE 6:**
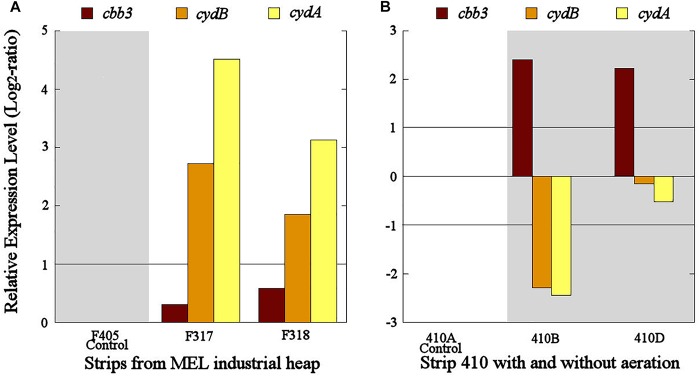
Transcriptional dynamics of *cbb3*, *cydA*, and *cydB* genes in *L. ferriphilum* from strips S317 (with aeration), S318 (with aeration), S405 (without aeration) **(A)** and S410 (before and after the aeration suspension) **(B)** of the industrial copper bioleaching heap. Gray area shows absence or low O_2_ availability.

The *cbb3* gene presented very different transcriptional dynamics in both studied industrial scenarios. In the industrial scenario of uninterrupted aeration condition, the *cbb3* gene did not show significant transcriptional differences between aerated (S317 and S318) and non-aerated strips (S405) (Figure 6A). However, an overexpression of the *cbb3* gene was observed at 14 (S410B) and 28 (S410D) days after aeration interruption of the industrial strip S410 (Figure 6B). On the other hand, *cydAB* genes maintained a congruent transcriptional dynamic in both industrial scenarios because they were significantly overexpressed in the aerated strips with respect to the non-aerated ones (Figure 6A) and underexpressed in all samples without aeration (S410B and S410D) (Figure 6B).

## Discussion

### Effect of the Oxygen Availability on *L. ferriphilum* Population and the Microbial Bioleaching Community

A very similar performance was observed in the cell growth and the ferrous oxidation rate at both repetitions in bioreactors tests with 3 and 10% of inlet-O_2_ (Figures 2A,B), suggesting that this specific difference in the O_2_ availability did not influence the growth and the oxidizing activity of this species. However, the differences in the cell growth curve and also the incomplete ferrous oxidation observed in cultures with 3–10% (45 and 51 h of culture) with respect to 23% of input-O_2_ strongly suggest a scarcity of final electron acceptor (O_2_) availability during the Fe^2+^ oxidation under those decreased O_2_ conditions (Figure 2B).

*Leptospirillum ferriphilum* was the predominant species in the bioleaching column and in the industrial strips (Figure 3). This result agrees with previous studies describing this species as one of the principal ferrous oxidizers in bioleaching systems (Demergasso et al., 2005; Galleguillos et al., 2009, 2013). The decrease in cell concentration of *L. ferriphillum* during the suspension of the aeration (OD62, 69, 97, and 104) and its subsequent increase observed after the oxygen resumption in the bioleaching column (OD76, 90, and 91) (Figure 3A) are both evidence of an inhibited growth of this species triggered by an oxygen starvation. Similar results of inhibited cell growth in response to low O_2_ availability has been also observed in *L. ferriphilum* from other bioleaching systems (Xingyu et al., 2010). Moreover, our results suggest that the growth of *L. ferriphilum* is probably affected after a sustained absence of oxygen in the system and not in an immediate short time as observed in Figure 3A that shows a drop in *L. ferriphilum* planktonic cell concentration only after 7 and 6 days after the first and second restriction of aeration, respectively.

The results of this study evidenced that *L. ferriphilum* as well as other species of the bioleaching community such as *Sulfobacillus* sp. and *A. thiooxidans* were negatively affected by the relatively low oxygen concentration when ranged from 2.5 to 0% of O_2_-out in the bioleaching column (Figure 3A). Similar results were also observed in the industrial strips without forced aeration (Figure 3B).

### Marker Genes as Indicators of Oxygen Availability in a Bioleaching System

The overexpression of *cbb3* and *cydAB* evidenced in the pure culture reactor with 23% O_2_ (Figure 4) would suggest that both terminal oxidases (cbb3 and bd-type) were performing as cytochromes with low-oxygen-affinity in *L. ferriphilum* DSM 14647 because they were overexpressed only at the higher O_2_ concentration (23% O_2_) (Bueno et al., 2012). However, only results of *cydAB* genes agree with those observed in the bioleaching column and in the industrial bioleaching strips. A pronounced and sustained under-expression with respect to the control was observed along all the time that aeration was suspended in the col2 (OD64, 0.19% O_2_; OD69, 2.55% O_2_; OD91, 1.88% O_2_; OD97, 0% O_2_; and OD104, 0% O_2_) (Figure 5), suggesting that the *cydAB* genes consistently presented transcriptional dynamics related to a terminal oxidase with low-oxygen-affinity in *L. ferriphilum*. These results differ from reported for this cytochrome complex in species such as *E. coli* (Borisov et al., 2011), *Rho. capsulatus* (Bueno et al., 2012) and *L. ferrooxidans* (Parro et al., 2007) where bd-type oxidase is described as a cytochrome with high-oxygen-affinity. Since none of the *L. ferriphilum* sequenced genomes have any described low-oxygen-affinity cytochromes it is possible that the bd-type complex could be fulfilling this function. In a number of organisms, the bd oxygen reductase is induced under O_2_-limited conditions as well as under other growth conditions that can be considered stressful, such as Fe deficiency (Borisov et al., 2011). However, in this study the bd complex was overexpressed at the same time that *L. ferriphilum* presented the best growth and ferrous oxidation rate so the observed transcriptional dynamics is most rather associated with an oxygen availability than a stress condition. On the other hand, Parro et al. (2007) reported that *cydAB* transcription rose in sectors of Rio Tinto with low oxygen saturation conditions (13 vs. 65% sat. O_2_ in water) but the scarcity of details in the transcriptomic data presented in that study did not allow us to perform a robust comparative analysis.

Transcriptional dynamics of *cydAB* genes in all industrial strips were also congruent with that observed in pure cultures and bioleaching column experiments (Figures 4–6). Both genes were always overexpressed and underexpressed when O_2_ concentrations were apparently sufficient or limiting, respectively. Based on these results, the bd-type cytochrome oxidase of *L. ferriphilum* consistently fulfills the function of a low-oxygen-affinity oxidase and validate *cydAB* genes as genetic markers associated to O_2_ availability for copper bioleaching systems. This finding differs from reported in *L. ferrooxidans* by Parro et al. (2007) who observed that transcription dynamics of bd-type cytochrome oxidase (*cydAB* genes) were induced at relatively low oxygen concentrations just like a cytochrome with high-oxygen-affinity. This could be another example of metabolic differences among related species triggered by adaptation processes, as it was exemplified above.

In contrast with the transcriptional dynamics of *cydAB* genes associated to the high O_2_-availability that was observed in all the experiments, the *cbb3* gene showed different transcriptional dynamics: (i) a gradual increase in the transcription level when forced aeration (O_2_) was abruptly cut off from the system, as in the case of the bioleaching column (Figure 5) and strip S410 (Figure 6B) and (ii) an increase in the transcription level in the reactor test with relatively high O_2_ concentration (23% O_2_) (Figure 4). The increased transcription of the *cbb3* gene observed at 7 and 14 days (OD97 and OD104) after the aeration suspension in the bioleaching column (Figure 5) and in the industrial strip 410 (Figure 6B), respectively, suggests that this terminal oxidase could be fulfilling the function of a high oxygen-affinity cytochrome when O_2_ conditions change abruptly in the system and it remains over time. This cbb3 function has been previously proposed for *L. ferriphilum* (Levicán et al., 2012) and for other species such as *B. japonicum and Rhodobacter sphaeroides* (Bueno et al., 2012). However, the *cbb3* gene does not seem to fulfill that function in *L. ferriphilum* DSM 14647, as it showed increased transcription levels at elevated oxygen concentrations in the reactor tests (Figure 4).

These results did not allow us to confirm the *cbb3* gene as a validated genetic marker associated solely to oxygen availability. Thus, it is possible to infer that the *cbb3* gene in *L. ferriphilum* is probably repressed or induced depending on oxygen availability and/or oxidative stress level. Aerobic respiration not only provides energy to the cell but, consequently, it also produces reactive oxygen species (ROS) such as hydrogen peroxide and hydroxyl radical that in excess trigger cellular damage and oxidative stress (Sigler et al., 1999). The role of terminal oxidases in preventing ROS production (Lindqvist et al., 2000; Poole and Cook, 2000; Giuffre et al., 2014) or in response to oxidative stress induced by excessive air sparging in continuous culture and by the growth on minerals (Christel et al., 2018) has been described. It is necessary to perform a deeper and more specific investigation to unravel the real function of this gene in the energy metabolism of *L. ferriphilum*. It is important to note that the *L. ferriphilum* type-strain (DSM 14647) is a laboratory- adapted strain and not one adapted to bioleaching industrial system as the case of *L. ferriphilum* from the bioleaching column (col2) and from the industrial strips from MEL heap. Adaptive differences among closely related strains has been widely reported (Brem et al., 2002). As an example, it has been reported that there are strains with (Llinás et al., 2006) and without (Gunasekera et al., 2017) nitrogen fixation ability within the *L. ferriphilum* species.

As far as we know, this is the first report of a transcriptomic approach related to the oxygen affinity of cytochromes oxidases in *L. ferriphilum* from which it was also possible to identify validated genetic markers for an industrial and operative copper bioleaching system.

## Conclusion

The results of this study allowed us to confirm that the oxygen availability is a key factor influencing the growth and gene transcription dynamics in *L. ferriphilum*. The bd-type cytochrome has a performance associated to a terminal oxidase with low-oxygen-affinity while cbb3-type cytochrome could be associated to different metabolic functions depending on the oxygen availability and oxidative stress level in this species. The consistent behavior of *cydAB* genes at different experimental scales provided the evidence to consider it as valid genetic markers associated to oxygen availability in this industrial heap bioleaching system.

## Data Availability

The raw data supporting the conclusions of this manuscript will be made available by the authors, without undue reservation, to any qualified researcher.

## Author Contributions

SM and CD conceived the presented idea, verified the analytical methods, and involved in the planning and supervision of the work. MC and SM developed the theory, performed the experiments, and wrote the manuscript with support from CD. SM performed both the identification of the candidate genes and the design of all primers for the gene-expression analysis by qPCR. MC carried out the gene expression experiments. DC involved in the design and supervision of the columns and reactor experiments. MC, SM, CD, and PG discussed the results and contributed to the final manuscript.

## Conflict of Interest Statement

The authors declare that the research was conducted in the absence of any commercial or financial relationships that could be construed as a potential conflict of interest.
