# Supplementary material for: Validation of Genetic Markers Associated to Oxygen Availability in Low-Grade Copper Bioleaching Systems: An Industrial Application
Source: Front Microbiol. 2019 Aug 9;10:1841. doi: 10.3389/fmicb.2019.01841 (PMC6695575; doi:10.3389/fmicb.2019.01841)

**Supplementary material**

**Supplementary Table 1.** Concentrations and purity indexes (260/280 and 260/230) of the RNA obtained from the samples of the pure cultures, experimental column and MEL strips. OD: Operational day of the column.

| Sample | | Identification Label | ng/μl | 260/280 | 260/230 |
| --- | --- | --- | --- | --- | --- |
| Reactor Exp-1 | 3% E1 | 107.7 | 1.82 | 1.07 |  |
| Reactor Exp-1 | 10% E1 | 61.8 | 1.84 | 0.90 |  |
| Reactor Exp-2 | 3% E2 | 43.5 | 1.77 | 0.94 |  |
| Reactor Exp-2 | 10% E2 | 42.9 | 1.95 | 1.38 |  |
| Reactor Exp-2 | 23% E2 | 45.5 | 1.77 | 0.93 |  |
| col1 | OD50 | 57.1 | 1.96 | 0.99 |  |
| col2 | OD62^(*)^ | 53.3 | 1.96 | 1.57 |  |
| col2 | OD64 | 160.8 | 1.79 | 2.18 |  |
| col2 | OD69^(**)^ | 58.6 | 1.78 | 1.41 |  |
| col2 | OD76 | 116.4 | 1.9 | 0.9 |  |
| col2 | OD90^(*)^ | 104.4 | 2.03 | 0.92 |  |
| col2 | OD91 | 127.4 | 1.89 | 0.42 |  |
| col2 | OD97 | 71.6 | 1.83 | 0.33 |  |
| col2 | OD104 | 48.3 | 1.74 | 0.3 |  |
| Strip 317 | S317 | 69.8 | 1.71 | 1.21 |  |
| Strip 318 | S318 | 27.6 | 1.6 | 1.14 |  |
| Strip 405 | S405 | 133.1 | 1.94 | 1.66 |  |
| Strip 410 | S410 A | 49.4 | 1.78 | 0.12 |  |
| Strip 410 | S410 B | 26.4 | 1.68 | 0.08 |  |
| Strip 410 | S410 C | 173.0 | 1.64 | 0.8 |  |
| Strip 410 | S410 D | 47.56 | 1.61 | 0.59 |  |

**Supplementary Figure 1.** Melting curve of the amplification of the *cbb3* gene of *L. ferriphilum*


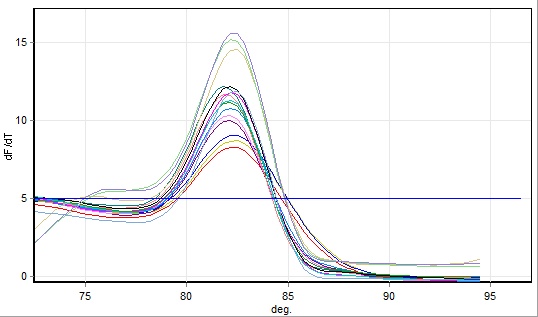


**Supplementary Figure 2.** Melting curve of the amplification of the *cydA* gene of *L. ferriphilum*
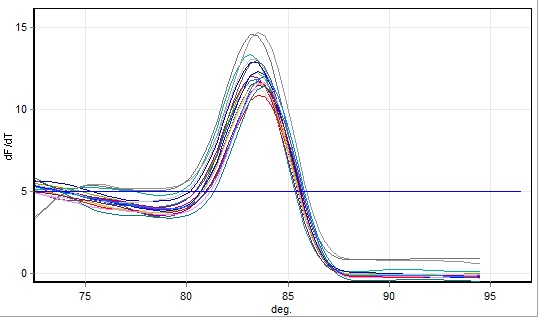


**Supplementary Figure 3.** Melting curve of the amplification of the *cydB* gene of *L. ferriphilum*


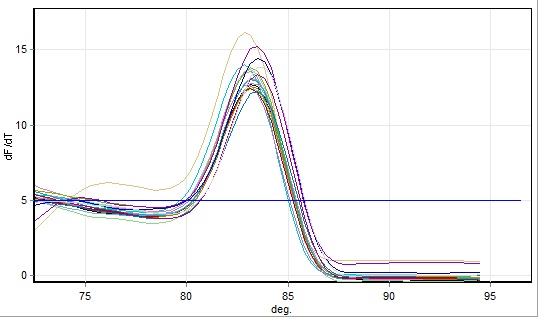


**Supplementary Figure 4.** Melting curve of the amplification of the *alaS* gene of *L. ferriphilum*


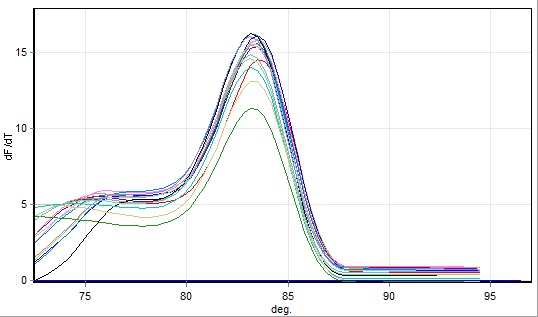


**Supplementary Figure 5.** Melting curve of the amplification of the 16S gene of *L. ferriphilum*


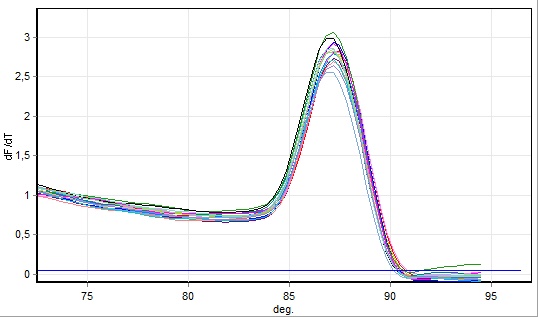

Supplement: Supplementary file 1 [file Data_Sheet_1.docx]
